# The impact of septic stimuli on the systemic inflammatory response and physiologic insult in a preclinical non-human primate model of polytraumatic injury

**DOI:** 10.1186/s12950-018-0187-6

**Published:** 2018-05-24

**Authors:** Diego A. Vicente, Matthew J. Bradley, Benjamin Bograd, Crystal Leonhardt, Eric A. Elster, Thomas A. Davis

**Affiliations:** 1 Department of Regenerative Medicine, Naval Medical Research Center, Silver Spring, MD USA; 2 Department of Surgery, Uniformed Services University of the Health Sciences & the Walter Reed National Military Medical Center, Bethesda, MD USA

**Keywords:** Inflammation, Trauma, Sepsis, Hemorrhage, Animal model, Cytokines, Immune dysregulation, Immunomodulation

## Abstract

**Background:**

Established animal trauma models are limited in recapitulating the pathophysiology of human traumatic injury. Herein, we characterize the physiologic insult and inflammatory response in two clinically relevant non-human primate (NHP) trauma models.

**Methods:**

Mauritian Cynomolgus Macaques underwent either a laparoscopic closed abdomen liver injury (laparoscopic 60% left-lobe hepatectomy) in an established uncontrolled severe hemorrhage model (THM), or a polytrauma hemorrhage model (PHM) involving combined liver and bowel injury, uncontrolled severe hemorrhage as well as an open full-thickness cutaneous flank wound. Fixed volume resuscitation strategies were employed in the THM and goal directed resuscitation was used in the PHM. Complete peripheral blood and critical clinical chemistry parameters, serum biomarkers of systemic inflammation, tissue perfusion parameters, as well as survival, were compared between the models throughout the 2-week study period.

**Results:**

NHPs in both the THM (*n* = 7) and the PHM (*n* = 21) demonstrated tissue hypoperfusion (peak lactate 6.3 ± 0.71 mmol/L) with end organ injury (peak creatinine 3.08 ± 0.69 mg/dL) from a similar liver injury (60% left hemi-hepatectomy), though the PHM NHPs had a significantly higher blood loss (68.1% ± 12.7% vs. 34.3% ± 2.3%, *p* = 0.02), lower platelet counts (59 ± 25 vs. 205 ± 46 K/uL, *p* = 0.03) and a trend towards higher mortality (90.5% vs. 33.3%, *p* = 0.09). The inflammatory response was robust in both models with peak cytokine (IL-6 > 6000-fold above baseline) and peak leukocyte values (WBC 27 K/uL) typically occurring around *t* = 240 min from the time of hepatic injury. A more robust systemic inflammatory response was appreciated in the PHM resulting in marked elevations in peak serum IL-6 (7887 ± 2521 pg/mL vs.1076 ± 4833 pg/mL, *p* = 0.02), IL-1ra (34,499 ± 5987 pg/mL vs. 2511 ± 1228 pg/mL, *p* < 0.00), and IL-10 (13,411 pg/mL ± 5598 pg/mL vs. 617 pg/mL ± 252 pg/mL, *p* = 0.03).

**Conclusion:**

This comparative analysis provides a unique longitudinal perspective on the post-injury inflammatory response in two clinically relevant models, and demonstrates that the addition of septic stimuli to solid organ injury increases both the hemorrhagic insult and inflammatory response.

## Background

The recent military conflicts in Iraq and Afghanistan have seen an unprecedented increase in survival of combat casualties despite increasing injury severity scores (ISS) and complex injury patterns [1, 2]. These improvements in combat casualty care can largely be attributed to progress in care on the battlefield, medical transport and resuscitation strategies [3]. As with civilian trauma, these severely injured combat casualties that survive the initial traumatic insult are at risk for developing an exaggerated inflammatory response with immediate and long term consequences such as sepsis, wound complications, heterotopic ossification (HO), invasive fungal infections, venous thromboembolic disease, multiple organ dysfunction syndrome (MODS), and multiple organ failure (MOF) [2, 4–12].

Despite extensive research, the underlying mechanisms for this dysregulated inflammatory response remains elusive both at the systemic and local tissue level. Attempts to mitigate the morbidity of this immune response through known pathways including blocking individual cytokines, co-stimulatory molecules, endothelial products, and complement cascade have yielded disappointing clinical outcomes in the face of promising preclinical results. This is in part due to the significant complexity and redundancy of the immune response to severe injury, which to date has not been adequately modeled in the preclinical setting by falling short on the model animal’s genetic similarity, degree of injury, goal directed critical care post-injury, and longitudinal evaluation of outcomes [10, 13]. Advances in preclinical modeling are required in order to better characterize and comprehensively differentiate the inflammatory response after various injury patterns, as well as to provide a better understanding for future interventions and clinical studies.

The nonhuman primate (NHP) is a highly valuable model which has significantly advanced our understanding of the physiological and immunological injury response patterns to acute trauma [10]. As part of a preclinical immunomodulation study, we have developed two preclinical NHP traumatic injury models including a closed abdomen, laparoscopic liver trauma and hemorrhage model (THM) with a fixed resuscitation protocol [14], as well as a closed abdomen polytrauma hemorrhage model (PHM) involving combined laparoscopic liver and bowel injury, uncontrolled hemorrhage as well as an open full-thickness cutaneous flank wound with subsequent goal directed resuscitation [15]. As previously reported, while the THM demonstrated a measurable systemic inflammatory response, only the few nonsurvivors developed end organ injury associated with limited resuscitation. Evolution of the PHM increased the mortality of the model, however, comparative analysis is required to better understand the complex interactions between polytraumatic injury, hemorrhagic shock, septic stimuli, and the inflammatory response. In developing this model the authors hypothesis was that a shift in the innate and adaptive immune responses would occur with the addition of septic stimuli and an associated increase in the systemic inflammatory response in the PHM despite the same liver injury in the THM consistent with a dysregulated response.

## Methods

### Animals

**Fig. 1:**
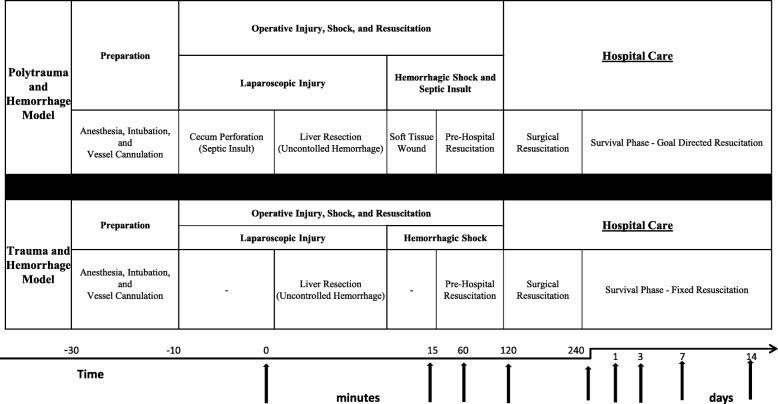
Time schematic of the polytrauma and hemorrhage model (PHM) and the trauma and hemorrhage model (THM). Non-human primates (NHP) in each group underwent similar pre-operative preparation, anesthesia induction and intubation, as well as insertion of central venous and arterial monitoring devices. In the THM, the NHPs underwent laparoscopic liver injury (60% left hepactectomy at *t* = 0 min) with 120 min of uncontrolled hemorrhage, pre-hospital resuscitation (20cm^3^/kg crystalloid at *t* = 15 min), surgical repair with liver repair and a 10cm^3^/kg warm whole blood transfusion (*t* = 120 min), as well as survival phase crystalloid post injury day 1 and 3 (20cm^3^/kg) and blood product resuscitation for anemia (Hb < 9). In contrast, the PHM NHPs underwent laparoscopic cecal injury prior to liver injury and back soft tissue injury prior to pre-hospital resuscitation. The post injury care in the PHM was goal directed with crystalloid resuscitation for evidence of poor perfusion (low urine output, elevated Cr or lactate), and blood transfusion for evidence of anemia (Hb < 7) and poor perfusion. Laboratory evaluations were performed 2 weeks prior to injury and at the listed time points in both models

Mauritian Cynomolgus Macaques (*Macaca fascicularis*) used in this study were adult (7.3 kg ± 0.15 kg) male (Worldwide Primates, Miami, Florida). The study protocol was reviewed and approved by the Walter Reed Army Institute of Research/Naval Medical Research Center (NMRC) Institutional Animal Care and Use Committee in compliance with all applicable Federal regulations governing the protection of animals in research. Animals were quarantined for approximately 45 days to acclimate to the animal facility. Prior to experimentation, animals were allowed free access to feed and water, though oral nutrition was withheld the night prior to surgery (12 h) to prevent aspiration during anesthesia. The methods for experimental protocols have been previously described at length [14, 15]. A schematic of each protocol’s schedule is depicted in Fig. 1, and a description is included below.

### Pre-injury preparation THM and PHM

The animals were transported to the operative suite on the morning of surgery and underwent sedation with telazol (Zoetis Inc., Kalamazoo, MI), peripheral IV placement, as well as endotracheal intubation with isoflurane mask induction and ventilation. Adequate anesthesia was administered and maintained with isoflurane via Datex Ohmeda S/5 ADU Carestation. ECG, pulse oximeter, end tidal carbon dioxide, rectal temperature probe, and urinary foley catheterization were established for intra-operative monitoring. FiO2 was set at 0.21 for the pre-hospital phase, and ventilation was adjusted to maintain physiologic pCO2 values (Apollo /Drǟger Medical, Telford, PA). At this time the animal’s abdomen and bilateral groins were shaved, prepped with a chlorhexidine/alcohol-based solution, and sterilely draped. A right femoral cut down was performed and a tunneled right central venous line (PORT-A-CATH, Smiths-Medical, Dublin, OH) and arterial catheter (Cordis, Johnson & Johnson, Miami Lakes, FL) were placed into the femoral vein and artery, respectively. The arterial catheter was connected to a hemodynamic monitoring system (Philips IntelliVue MP70, Philips Electronics North America Corporation, Andover, MA) for continuous monitoring of arterial pressures and intra-operative lab draws. Attention was then turned to prepare the laparoscopic abdominal injuries. Access to the peritoneal cavity was performed via Hassan technique and a 12 mm trocar (ENDOPATH XCEL, Ethicon, Somerville, NJ) was left in place. Pneumoperitoneum was established with CO_2_ to an intra-abdominal pressure of 10–12 mmHg. A 10 mm 30 degree laparoscope was inserted through the port and peritoneal cavity was examined. A 5 mm trocar was placed in the left hypogastric region, and a second 5 mm trocar was placed in the right hypogastric region.

### Injury phase THM

Under laparoscopic visualization, animals in the hemorrhage model underwent a non-anatomic 60% left lobe liver resection creating uncontrolled hemorrhage which was designated time zero (*t* = 0 min). The pneumoperitoneum was then evacuated, all laparoscopic ports were removed, and the port sites were temporarily closed with 2–0 nylon sutures to achieve abdominal wall continuity.

### Injury phase PHM

Under laparoscopic visualization, animals in the PHM underwent a 4 cm anti-mesenteric cecal injury to allow for abdominal sepsis. A 60% left lobe liver injury was then created (t = 0 min) and temporary abdominal closure was performed as described above. The animals in this model additionally underwent a right flank standardized circular (18.85cm^2^) full-thickness soft tissue injury down to the level of the muscle fascia.

### Pre-hospital care THM and PHM

At *t* = 15 min, pre-hospital resuscitation was initiated with normal saline resuscitation (20 mL/kg) for both the hemorrhage alone and polytrauma groups [16]. Anesthetic, cardiovascular, and pulmonary monitoring were continued throughout the 120 min pre-hospital phase.

### Resuscitation phase/hospital care THM

Surgical resuscitation was initiated at the start of the hospital phase (*t* = 120 min) with a midline laparotomy. The hepatic injury was exposed and hemostasis was achieved by suture ligation, cautery, and Surgicel (Ethicon, Somerville, NJ). All intra-peritoneal blood and the resected liver segment were removed and the volumes were recorded as total hemorrhage volume, percent blood loss, and percent hepatectomy. [17] At *t* = 120 min the THM NHPs underwent continuous monitoring, oxygen therapy, blood-type matched and leukocyte- reduced blood transfusion at 10 mL/kg, active rewarming. The abdomen was closed and at *t* = 240 min the last blood draw was withdrawn and the arterial and venous catheters were removed, anesthesia was weaned, and the animals were extubated.

### Resuscitation phase/hospital care PHM

The animals in the PHM also underwent surgical resuscitation at *t* = 120 min with similar hepatic repair and injury measurements as described in the THM. Once hemostasis was achieved the cecal perforation was repaired in two-layer closure. In addition, the peritoneal cavity was copiously irrigated and all fecal contamination was removed. The abdomen was closed at this time. At t = 120 min the PHM NHPs also underwent continuous monitoring, oxygen therapy, blood-type matched and leukocyte- reduced blood transfusion at 10 mL/kg, active rewarming. After the initial blood transfusion, NHPs underwent goal directed resuscitation to correct MAPs < 40 mmHg, base excess < − 4, and/or lactate > 4, and decreased urine output (< 0.5 mL/kg) with crystalloid resuscitation. NHPs with hemoglobin < 7 g/dL were resuscitated with blood transfusions. Once the animals were resuscitated, the flank soft tissue wound was then dressed (Hydrofera BLUE®, Hollister Wound Care, Libertyville, IL), and the arterial catheter was removed. The venous port was tunneled and remained in place with the port secured in subcutaneous space. The animals were then placed in a protective jacket (Lomir Biomedical Inc., Quebec, Canada), and they were extubated after weaning anesthesia.

### Antibiotics THM and PHM

At the initiation of surgical resuscitation, animals in both groups received a dose of broad-spectrum antibiotics (enrofloxacin 5 mg/kg [Bayer HealthCare LLC, Shawnee Missino, KS]) and metronidazole 50 mg/kg [Henry Schein, Melville, NY].

### Survival phase THM

The animals in the THM group were recovered in an “intensive care unit” setting with 24 h of continuous monitoring by trained research and veterinary staff. Animals were administered buprenorphine (0.1–0.3 mg/kg) every 4 h as needed for pain, peripheral intravenous normal saline (20 mL/kg) on post-injury day 1, 2, and 3, and a blood transfusion (10 mL/kg) for anemia (Hb < 9 g/dL). If clinically stable after 24 h, the animals were returned to their pre-operative housing. At least twice daily evaluations were performed in conjunction with veterinary staff, and euthanasia was performed based on adverse clinical states and complications, response to resuscitation, response to pain medications, and laboratory parameters to include indices of fulminant renal failure. If criteria were met, animals were euthanized with pentobarbital (Fatal-Plus; Vortech Pharmaceuticals, Dearborn, MI). All surviving animals were euthanized according to protocol on post-injury day 14 followed by a complete necropsy evaluation.

### Survival phase (PHM)

In the PHM group, the animals were also recovered in an ICU setting, but for at least 72 h with laboratory staff present for continuous monitoring and goal directed resuscitative interventions with crystalloid infusion to correct signs of poor perfusion (low urine output, elevated Cr or lactate) or blood transfusions to correct signs of anemia (Hb < 7 g/dL) and poor perfusion. Laboratory evaluation was performed every four hours as needed to evaluate the response to resuscitation while in the ICU setting. Pain management was performed with sustained release transdermal fentanyl patch (25mcg/hr. placed at *t* = 240 min) and buprenorphine (0.01–0.03 mg/Kg IM every 6 h as needed). The soft tissue wound was examined and dressed every 5 days. If assessed clinically stable, animals were advanced to a regular diet and returned to their pre-operative housing. Euthanasia with pentobarbital was administered at the veterinarian’s discretion or if the animals demonstrated signs of severe disability, severe infection, uncontrolled pain, or showed clinical/laboratory signs of deterioration and failed to respond to resuscitation. On post-injury day 14, surviving animals were euthanized and underwent a complete necropsy evaluation.

### Laboratory and survival analysis (THM and PHM)

For the purposes of this study, laboratory analysis were contrasted between the two models’ comparable data points which included the blood collected at 2 weeks pre-injury as well as *t* = 0, 15, 60, 120, and 240 min, as well as on post-injury days 1, 3, 5, 7 and 14. Blood was analyzed for complete blood cell count, arterial blood gas, and basic metabolic profile. In addition, the serum profiles for a panel of 14 cytokines and chemokines (IL-6, IL-1ra, IL-10, G-CSF, MCP-1, IL-1β, IFN-γ, TNF, GM-CSF, IL-15, MIP-1α, IL-8, IL-2, TGFα) was determined using multianalyte bead based profiling (Luminex; NHP 14-plex; Millipore, Billerica, MA). Survival time was recorded according to time of intra-operative death (t = 0 to *t* = 240 min) or post-injury day.

### Statistical analysis

Comparable variables from the two models including markers of injury, mortality, cytokine levels, and peripheral leukocyte counts were analyzed with IBMSPSS® Statistics (version 23.0, Chicago, IL, USA). Percent blood loss was calculated using the formula: [blood loss volume (ml)/total estimated blood volume (TEBV)] × 100. TEBV was calculated based on 5.4% of the animal’s weight according to previously published data on blood volume in Cynomolgus Macaques [17]. Percent hepatectomy was calculated using the formula: [liver cut length (cm)/full liver length (cm)] × 100. An Injury Severity Score (ISS) was calculated for every animal in each group [18].

## Results

The physiologic parameters and markers of injury have been previously reported for both the THM [14] and PHM [15]. Both models entail damage control resuscitation following severe hemorrhage. In this comparative analysis, the percent hepatectomy and physiologic insult (hypotension, maximum creatinine and lactate) was similar between animals in the THM (*n* = 7) and PHM (*n* = 21), as seen in Table 1. However, despite having similar percent hepatic injuries, animals in the PHM had a significantly larger degree of intra-abdominal hemorrhage (68.1% ± 12.7% vs 34.3% ± 2.33% blood loss; *p* = 0.02), and lower platelet counts (59 K ± 25 K/μl vs 205 K ± 46 K/ul; *p* = 0.03). It should be noted that the addition of the soft tissue injury in the PHM had minimal contribution to the blood loss.

**Table 1:** Markers of injury compared between the polytrauma and hemorrhage model (PHM) and the trauma and hemorrhage model (THM).

Pre-Hospital and Hospital Phase Markers of Injury	Model		
	PHM (*n* = 21)	THM (*n* = 7)	p
Injury Severity Score	21	16	–
Left Hepatectomy (%)	60.6 ± 4.3	55.9 ± 3.76	0.42
Blood Loss (%)	68.1 ± 12.7	34.3 ± 2.33	0.02
Minimum MAP(mmHg)	24.6 ± 1.92	34.5 ± 6.5	0.22
Minimum Temperature(°F)	90.0 ± 3.66	94.3 ± 0.38	0.26
Minimum pH	7.1 ± 0.1	7.2 ± 0.1	0.63
Minimum Platelets (K/*u*L)	59 ± 25	205 ± 46	0.03
Experimental Injury Values			
Cr (max)	1.8 ± 0.36	3.08 ± 0.69	0.17
Lactate (max)	6.3 ± 0.71	4.88 ± 0.62	0.16

**Fig. 2:**
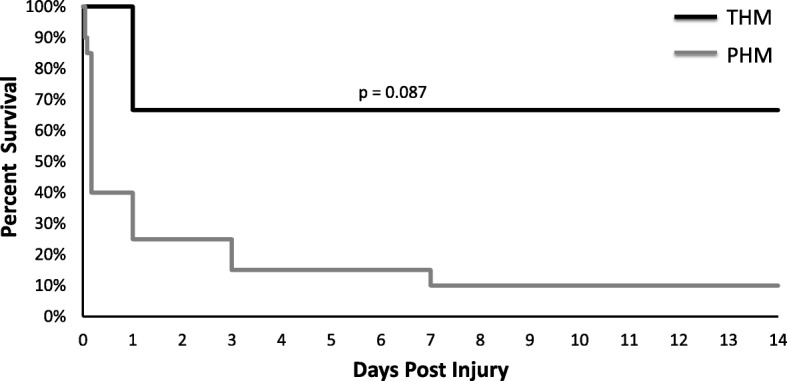
Kaplan-Meier survival curves demonstrating trend towards increased mortality in polytrauma hemorrhage model (PHM) and hemorrhage model (THM)

The animals in the PHM had a trend towards higher mortality (*p* = 0.087) as seen in Fig. 2 despite goal targeted resuscitation for the first 72 h following injury to correct signs of poor perfusion. The non-survivor NHPs in the THM were euthanized on post injury day-1 upon demonstrating decreased responsiveness and progressive renal failure (Cr > 4.5 at time of euthanasia) on the fixed resuscitation protocol. The survivor NHPs demonstrated limited evidence of injury and were alert, ambulatory, normotensive, and tolerating a diet 36 h post-injury. The non-survivor animals in the PHM included intra-operative deaths (*n* = 3) and euthanasia prior to day-1 post injury (*n* = 9) associated with hemorrhagic shock, cardiovascular collapse, or failure to wean from ventilator support. Euthanasia on post injury day-3 (*n* = 2) was performed for respiratory failure. Finally euthanasia performed on post injury day-7 (*n* = 1) was performed for progressive clinical signs of distress associated intra-abdominal sepsis, which was confirmed on necropsy. The survivor NHPs in the PMH, in contrast to the THM, demonstrated prolonged recoveries with limited mobility and diet tolerance for 2–3 days after injury.

**Fig. 3:**
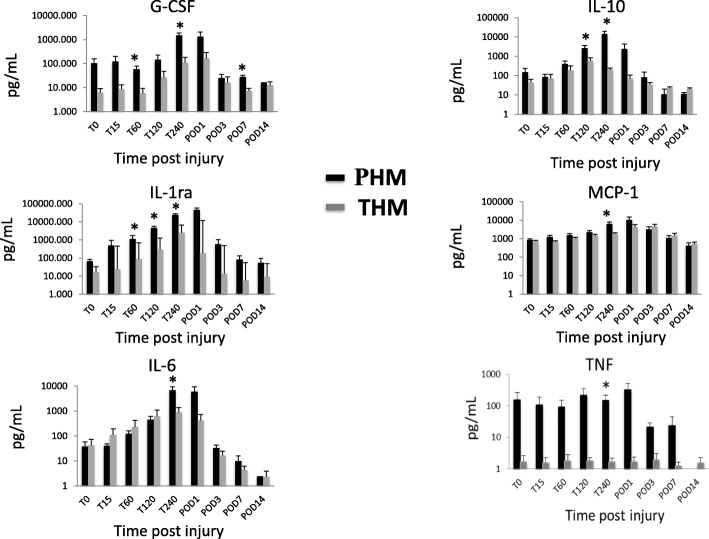
Cytokine and chemokine levels depicted over time of 6 cytokines and chemokines quantified in serum using multi-analyte Luminex profiling at various time points post liver injury. Levels at each time point of both the THM and PHM were plotted as mean ± SEM, and compared by Mann–Whitney U test. (*p ≤ 0.05)

The magnitude of the systemic inflammatory response to the different trauma injury patterns in THM (liver injury + hemorrhage) and PHM (liver, bowel and soft tissue injury + hemorrhage) was markedly different between the two models as seen in Table 2 and Fig. 3. The maximum recorded values in the post-injury period and the respective fold change from pre-injury baseline for markers of systemic inflammation in the post-injury period demonstrated that serum IL-6, IL-1ra, IL-10, G-CSF, MCP-1, TNF, all had at least a 10 fold change from baseline in one or both of the models. The inflammatory markers IL-1β, IFNγ, GM-CSF, IL-15, MIP-1α, IL-8, IL-2, and TGFα were non-detectable, negligible, or did not significantly change from baseline. Overall, the PHM induced a more severe systemic inflammatory response as compared to the THM group.

**Table 2:** Maximum recorded cytokine values as well as fold change from baseline (2 weeks pre-injury) of systemic cytokines throughout the post-injury period (t = 0 to post-injury day 14) from the trauma and hemorrhage model (THM) and polytrauma and hemorrhage model (PHM)

	Maximum Recorded Serum Level				
	Polytrauma and Hemorrhage Model		Trauma and Hemorrhage Model		
	Maximum (pg/mL)	Fold change	Maximum (pg/mL)	Fold change	*P*-value
IL-6	7887 ± 2521	6149	1076 ± 483	1159	0.015
IL-1ra	34,498 ± 5987	2286	2511 ± 1229	725	0.000
IL-10	13,411 ± 5598	785	617 ± 252	50	0.034
G-CSF	1652 ± 1912	51	201 ± 192	15	0.003
MCP-1	8419 ± 2297	19	4064 ± 1140	4.5	0.104
IL-1β	27 ± 71	19	0.5 ± 0.2	1.0	0.103
IFNγ	40 ± 126	18	4.7 ± 6.8	2.6	0.208
TNF	289 ± 558	11	2.6 ± 2.0	1.9	0.040
GM-CSF	30 ± 41	7.4	1.4 ± 1.5	5.1	0.006
IL-15	19 ± 18	3.8	13 ± 4.4	10.5	0.092
MIP-1α	47 ± 52	3.5	12 ± 16	1.4	0.016
IL-8	2056 ± 2190	3.0	1280 ± 737	1.3	0.140
IL-2	19 ± 22	2.1	10.4 ± 5.5	1.5	0.112
TGFα	50 ± 87	2.1	37 ± 15	2.0	0.534

Figure 3 demonstrates the cytokine levels throughout the post-injury period of those cytokines which were detectable and varied significantly from baseline. The PHM animals had a significantly larger cytokine elevation as compared to the THM typically around *t* = 240 min. However, significant differences occurred earlier between the two models at *t* = 60 min with IL-1ra and G-CSF, as well as at *t* = 120 min with IL-10. All cytokines began to down trend towards baseline by day-3 post injury.

**Fig. 4:**
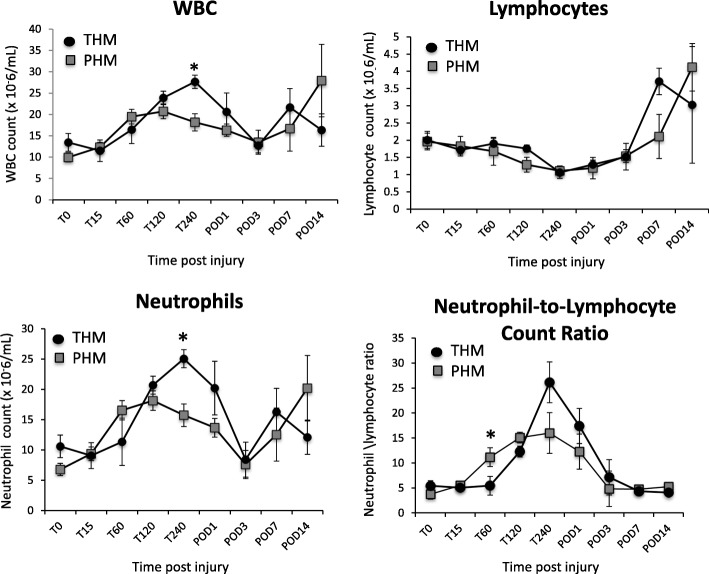
Changes in systemic hematological parameters (WBC, neutrophil and lymphocyte counts, and neutrophil-lymphocyte count ratio) immediately before the initiation of liver injury and at various time points post injury. Individual points were plotted as mean ± SEM. The difference between the THM and PHM groups was tested using Mann–Whitney U test. (*p < 0.05)

The inflammatory response as depicted by the peripheral total and differential leukocyte counts is seen in Fig. 4. Overall there is a general increase in circulating neutrophils and decrease in lymphocytes through 240 min in both models, with fluctuations around baseline thereafter. Both the WBC and neutrophil counts are significantly higher in the THM animals at t = 240 min. The neutrophil:lymphocyte ratio is higher in the PHM model at t = 60 min.

## Discussion

Severely injured polytrauma patients are at risk for shock, immune dysregulation, MODS, and MOF. The addition of septic insult in trauma patients and experimental animal models compounds the inflammatory response, and can increase both the morbidity and mortality of trauma [19–22]. Clinically effective strategies to mitigate the deleterious effects of this inflammatory response are principally centered on supportive care, and improvements in modeling this inflammatory response are required in order to better understand and target inflammatory mediators. In this study, we characterized and compared the physiologic injury and the systemic inflammatory response in two separate NHP trauma models, and demonstrated that the addition of septic stimuli to trauma and hemorrhage caused a distinct end organ injury pattern as well as an increased inflammatory response, hemorrhage, and subsequent morbidity and mortality despite improvements in resuscitation.

Both the THM and PHM animals developed evidence of renal injury and tissue hypoperfusion. The renal injury and mortality seen on post-injury day-1 of the THM, as previously described by our group, was more likely associated with inadequate resuscitation rather than systemic inflammation associated multiple organ dysfunction syndrome [14]. In contrast, PHM NHPs demonstrated clinical observations and pathology results consistent with acute respiratory distress syndrome [15]. It is postulated that the multiple inflammatory stimuli in these animals resulted in more significant distant end-organ damage, especially in the respiratory system, which ultimately lead to a trend towards higher mortality.

Interestingly, the NHPs in the PHM experienced significantly larger hemorrhage despite having a similar liver injury as the THM. This hemorrhage was associated with a relative thrombocytopenia, but not with other predisposing factors such as a significantly lower pH or temperature. Though we did not measure coagulation parameters in this study, we suspect that the thrombocytopenia and larger hemorrhage in the PHM was secondary to a sepsis induced coagulopathy [20, 22–31] with immune mediated endothelial activation and induction of coagulation cascade [23, 25, 26, 32, 33]. The coagulopathy of sepsis has been studied in detail, and specifically demonstrated in two prior NHP models [30, 34]. In a lethal intravenous *E. coli* NHP model, *Taylor* demonstrated that septic stimuli initiated not only an inflammatory response (elevated IL-6, TNF, and C5b-9), but also an early consumptive coagulopathy through endothelial activation (elevated Tissue Factor, Thrombomodulin, Endothelial Linked Adhesion Molecules) and induction of the coagulation cascade (depressed Fibrinogen) [34]. Similar findings were also seen in a NHP peritonitis model in which the septic stimuli resulted in a significant inflammatory response and drop of both fibrinogen and platelets within 2 h of initial laparotomy [30]. Recently, a traumatic shock rabbit model demonstrated that while hepatic injury and hemorrhage alone induce hypercoagulable findings on thromboelastography, the addition of bowel injury to the hepatic injury pattern induced a hypocoagulable state [31]. In keeping with the reported literature, the increased hemorrhage, thrombocytopenia, and mortality associated with the septic insult in the PHM compared to THM reinforces the interconnected and interdependent immune, reticuloendothelial, and hemostatic systems, particularly in the setting of polytrauma.

To our knowledge, this is the first study to characterize and contrast the longitudinal inflammatory response in two separate NHP trauma models to post-injury day 14. The inflammatory response in all detectable cytokines in both the THM and PHM typically peaked around *t* = 240 min, and this response persisted through post-injury day 1, but then proceeded to trend down by day 3. This mixed early peak in both pro-(IL-6 and TNF) and anti-inflammatory (IL-10) cytokines between time of insult (trauma, hemorrhage, and/or sepsis) and day 1 has been seen in inflammatory stimuli animal models as well as clinical sepsis studies [21, 29, 30, 35–43]. This mixed immune response has recently demonstrated in the clinical setting by Hazeldine et al. with significant elevations of both pro- and anti-inflammatory signaling within an hour of trauma [12]. Specifically reviewing the literature for comparable longitudinal data on IL-6, IL-10, and TNF as common markers of inflammation, Table 3 depicts available data from relevant animal models (NHP and Swine) and clinical studies wherein early (within 24 h) peak cytokine levels are seen in almost all studies. Cytokine peaks occurring beyond 24 h were only demonstrated in animal models with a persistent septic stimulus [20]. Further review of the literature revealed that clinical studies with delayed cytokine peaks were seen in patients with trauma associated with MODS/MOF [44–46], abdominal aortic aneurysm repair associated with MOF [47], trauma associated with ARDS [44], sepsis [21], and serial debridements of combat wounds [48]. Given the early mortality in the PHM associated with respiratory failure, it is likely that delayed peaks in inflammatory markers were likely missed, and we suspect that ventilator support in the hospital phase for these NHPs would have yielded additional longitudinal data that may have demonstrated delayed peaks in the inflammatory cytokines [15].

**Table 3:** Clinical studies and animal models evaluating longitudinal cytokine response to trauma

		IL-6		IL-10		TNF	
Study (reference #)	Injury/Insult, n	Mean Peak (pg/mL)	Time Point	Mean Peak (pg/mL)	Time Point	Mean Peak (pg/mL)	Time Point
Kubiak 2011 [20] Animal (Insult): Swine (Trauma, IRI, Sepsis) Survival: 48 h	*Trauma (laparotomy) + IRI (SMA clamp × 30 min) + Sepsis (fecal clot) with passive drainage at 12 h. n = 5*	23,702	36 h	163	6 h	852	48 h
Kinasewitz 2000 [30] Animal(Insult): NHP (Trauma, Sepsis) Survival: 14 days	*Trauma (laparotomy), Sepsis (E coli fibrin clot peritonitis). n = 5 (non-survivor)*	~ 22,000	4 h			~ 31,000 (death)	2 h
	*Trauma (laparotomy), Sepsis (E. coli fibrin clot peritonitis). n = 4 (signs of sepsis)*	~ 22,000	4 h			~ 12,000 (sepsis)	2 h
	*Trauma (laparotomy), Sepsis (E. coli fibrin clot peritonitis). n = 4 (well appearing)*	~ 10,000	4 h			~ 12,000 (well)	2 h
Martin 1997 [21] Clinical Study (Sepsis vs. Trauma and Survivors vs. Nonsurvivors) and (Sepsis vs. Trauma and Hemorrhage)	*Septic Shock (mean APACHE II score = 27) non-survivors n = 14*	15,627	arrival			34	9th blood draw
	*Septic Shock (mean APACHE II score = 27) survivors n = 11*	~ 4000	arrival			~ 20	3rd blood draw
	*Trauma (mean ISS = 27) non-survivors n = 7*	600	8th blood draw			30	blood draw 13
	*Trauma (mean ISS = 27) survivors n = 53*	~ 200	peak			~ 20	blood draw 11
	*Trauma (mean ISS not reported) + Hemorrhagic Shock n = 8*	420	2nd blood draw			~ 30	8th blood draw
Jastrow 2009 [40] Clinical Study (Trauma +/− MOF)	*Trauma (mean ISS 26) with MOF. n = 11*	7179	2–6 h	239	2–6 h	211	2–6 h
	*Trauma (mean ISS 25) with nonMOF. n = 37*	427	6–10 h	15	2–6 h	0	2–6 h
PHM Animal: NHP (Trauma, Hemorrhage, and Sepsis) Survival: 14 days	*Trauma (laparoscopic liver/bowel injury, flank soft tissue injury) + intra-abdominal hemorrhage/ sepsis × 2 h. n = 20*	6860	4 h	13,519	2 h	321	24 h
THM Animal: NHP (Trauma and Hemorrhage) Survival: 14 days	*Trauma (laparoscopic liver injury) + Hemorrhage (uncontrolled hemorrhage × 120 min)n = 5*	1168	4 h	588	2 h	2	60 min
Sheppard 2017 [43] Animal: NHP (Trauma and Hemorrhage) Survival: 24 h	*Trauma (Lapartomy and Femur Fracture) + Decompensated Hemorrhage (pressure controlled)*	~ 750	6 h	~ 450	< 6 h		
	*Trauma (Laparotomy and Femur Fracture) + 60 min Hemorrhage (Pressure Controlled)*	~ 400	6 h	~ 650	< 6 h		
Deitch 1996 [41] Animal: NHP (Trauma and Hemorrhage vs. Trauma) Survival: 8 h	*Trauma (neck dissection and partial removal of clavicle) + Hemorrhage (40 mmHg × 3 h or reached -5 mEq Base Excess). n = 5*	~ 350	~ 4 h				
	*Trauma (neck dissection and partial removal of clavicle)*	~ 100	~ 8 h				
Namas 2009 [38] Clinical Study (Trauma)	*Human Trauma (mean ISS 24) + Hemorrhagic Shock n = 4 (non-survivors)*	202^a^	within 6 h	33^a^	within 6 h	4^a^	within 6 h
Baker 2012 [35] Animal: Swine (Trauma vs. Hemorrhage vs. Trauma and Hemorrhage) Survival: 300 min	*Trauma (femur fracture and lung contusion) + Hemorrhage(20-30 mmHg × 60 min). n = 6*	~ 170	2 h	~ 15	baseline	133	baseline
	*Trauma (femur fracture and lung contusion). n = 5*	~ 170	5 h	~ 100	45 min	~ 300	60 min
	*Hemorrhage(20-30 mmHg × 60 min)n = 5*	~ 150	3 h	~ 41	< 15 min	~ 290	45 min
Namas 2009 [38] Animal (insult): Swine (Trauma and Hemorrhage vs. Hemorrhage) Survival: 2 h after resuscitation	*Trauma (anterolateral thoractomy and pericardial window) + Hemorrhage (MAP < 30 mmHg × 10 min, MAP < 20 mmHg for 10 s, or MAP>/= 30 × 90 min). n = 4 (non-survivors)*	~ 90	90 min	~ 15	90 min	~ 170	90 min
	*Hemorrhage (MAP < 30 mmHg × 10 min, MAP < 20 mmHg for 10 s, or MAP >/= 30 × 90 min). n = 3 (non-survivors)*	~ 1	baseline	~ 20	60 min	~ 100	baseline
	*Trauma (anterolateral thoractomy and pericardial window) + Hemorrhage (MAP < 30 mmHg × 10 min, MAP < 20 mmHg for 10 s, or MAP>/= 30 × 90 min). n = 7 (survivors)*	~ 40	90 min	~ 1	baseline	~ 240	90 min
	*Hemorrhage (MAP < 30 mmHg × 10 min, MAP < 20 mmHg for 10 s, or MAP >/= 30 × 90 min). n = 9 (survivors)*	~ 30	baseline	5	baseline	~ 600	0 min

Literature review of animal and clinical studies comparing trauma +/− sepsis +/− hemorrhagic insults

~Serum cytokine level (pg/mL) approximated from article’s figure

*NHP* nonhuman primate, *NMRC* Naval Medical Research Center, *PHM* Polytrauma and Hemorrhage Model, *THM* Trauma and Hemorrhage Model, *IRI* Ischemia Reperfusion Injury, *SMA* Superior Mesenteric Artery

^a^mean cytokine value within first 6 h of injury

As has been previously reviewed [49], compared to solid organ injury and uncontrolled hemorrhage models such as the THM, the pressure controlled hemorrhage and trauma models listed in Table 3 result in a relatively limited inflammatory response [35, 38, 41, 43]. The addition of septic stimuli and soft tissue trauma in the PHM significantly increased the inflammatory response with a greater than 6100-fold elevation in IL-6 levels from baseline in the PHM, compared to the only 1100-fold increase in the THM. Preclinical studies favor sepsis as the major driver of the inflammatory response [36, 50], and clinical studies have shown IL-6 elevation has been tied to sepsis in trauma [21, 47, 51–57]. Table 3 also demonstrates that sepsis alone or the addition of sepsis to trauma results in a more pronounced inflammatory response than trauma alone, hemorrhage alone, or trauma with hemorrhage [20, 21, 30]. The highest IL-6 peaks (> 15,000 pg/mL) in Table 3 occurred in animals with persistent septic stimuli [20, 30] and non-surviving patients suffering from septic shock [21]. As seen in our PHM, relatively lower cytokine levels seen within the first 24 h in NHPs who underwent intra-peritoneal sepsis × 2 h with subsequent peritoneal washout and repair of bowel injury were reflected in other studies in which the animals were able to clear their septic stimuli [30] and patients who were able to recover from septic shock [21]. Though comparing cytokine levels between studies can be challenging given inconsistencies in analysis technique and variation in response to inflammatory stimuli across species [13], it is interesting that the IL-6 threshold (> 4110 pg/mL) predictive of MOF in trauma patients demonstrated by Jastrow et al., also distinguishes the IL-6 levels between the PHM and THM, as well as in the results of other studies seen in Table 3 contrasting trauma alone vs. sepsis ± trauma in both animal models and clinical studies. This may be suggestive that this IL-6 threshold distinguishes early peak inflammation in response to certain inflammatory stimuli that predicts subsequent immune dysregulation. Comparing the reported TNF and IL-10 levels to the literature, as seen in Table 3, demonstrates that the levels in the PHM cross the MOF predictive threshold of TNF and IL-10 described by Jastrow [40], however, the differential response per inflammatory stimuli in other studies is not as consistent as is seen with IL-6.

The leukocyte data presented in this study reveals the expected post-injury changes associated with severe trauma including leukocytosis, neutrophilia, and relative lymphocytopenia in both the THM and PHM [58–67]. Interestingly, despite a more significant systemic cytokine response, the NHPs in the PHM had a lower WBC and neutrophil count at *t* = 240 min, and a higher neutrophil:lymphocyte ratio at *t* = 60 min. The neutrophil: lymphocyte ratio has emerged as an important prognostic factor in inflammatory conditions such as those found in critically ill patients and cancer patients [66, 68]. The PHM’s higher neutrophil:lymphocyte ratio in addition to the relative neutropenia and leukopenia compared to the THM are consistent with the reported literature that increased tissue trauma and the intra-abdominal sepsis induced stress-induced lymphocyte apoptosis [69] as well as greater neutrophil and lymphocyte sequestration [59, 66, 70–72] both at the site of sepsis and in distant organs such as the lungs [15] seen in prior studies. These early alterations in the balance of innate/adaptive immune responses in trauma imply long term consequences of immune dysregulation and increased mortality [73].

## Conclusions

Physiologically relevant and highly reproducible preclinical animal models of polytraumatic injury are required to reproduce the complex and heterogeneous pathobiology of human polytraumatic injury and to develop novel therapeutic strategies. In an attempt to move from the bench to the bedside with the aim to test candidate therapeutic immunomodulatory agents that may dampen the early trauma-induced inflammatory/immune response, we have evaluated the inflammatory response in two preclinical NHP trauma models. Analysis of comparable variables between the two models demonstrated that the addition of septic stimuli in the PHM leads to a larger hemorrhage and a mixed inflammatory systemic cytokine response, as well as early alterations in innate/adaptive immune response compared to the THM. This study’s novel longitudinal comparison of systemic cytokines in the two separate models demonstrated that the addition of sepsis crosses predictive threshold of IL-6, IL-10, and TNF for immune dysregulation. Further, this analysis supports clinical studies demonstrating acute traumatic coagulopathy with polytrauma, as well as prompts further exploration of hemostatic complications of even transient septic stimuli in trauma patients. Future efforts in model development will focus on extended ventilator support phase for recovery, as well as expanded coagulation, systemic biomarkers, tissue immunohistochemistry, and flow cytometric studies to further characterize the effects of trauma and sepsis and potentially elucidate therapeutic targets to mitigate the inflammatory response of trauma.
